# Follow-up of children and adolescents with special health care needs: Results from the KiGGS study 2003-2012

**DOI:** 10.17886/RKI-GBE-2017-063

**Published:** 2017-12-13

**Authors:** Elvira Mauz, Roma Schmitz, Christina Poethko-Müller

**Affiliations:** Robert Koch Institute, Department of Epidemiology and Health Monitoring, Berlin

**Keywords:** CSHCN, CHRONIC DISEASE, SELF-ASSESSMENT, CHILDREN AND ADOLESCENTS, COHORT STUDY

## Abstract

The incidence of chronic diseases in children has risen sharply throughout the world. Regardless of the particular diagnosis, chronically ill children and their families are faced with particular challenges. The transition to adulthood leads to changes in the conditions in which care is provided and the responsibilities that are associated with it. In this paper we used KiGGS cohort data to investigate the odds that chronically ill children and adolescents have of no longer feeling chronically ill or facing health impairments in young adulthood. Furthermore, it investigates the factors that are associated with an unfavourable transition from childhood into young adulthood.

The analysis employs a data subgroup sourced from the first two waves of the KiGGS cohort – the longitudinal component of the German Health Interview and Examination Survey for Children and Adolescents (KiGGS) conducted by the Robert Koch Institute. The population-based KiGGS baseline survey was carried out between 2003 and 2006 as an examination and interview survey of 17,641 children and adolescents aged between 0 and 17. KiGGS Wave 1 – its first follow-up – was conducted between 2009 and 2012 via telephone. The KiGGS baseline survey used the German translation of the Children with Special Health Care Needs (CSHCN) screener as part of the written questionnaire provided to parents. This formed part of a generic approach towards identifying chronically ill children that applies irrespective of the diagnosis in question. KiGGS Wave 1 used a generic query to gather data on chronic illnesses using two questions taken from the Minimal European Health Module (MEHM); by this stage, the participants had reached adulthood.

The information provided by 3,243 KiGGS cohort participants (who were aged between 18 and 24 at the time of KiGGS Wave 1) was analysed for indications of the presence or absence of a chronic disease among children and adolescents aged between 12 and 17. This was done using data gathered using the CSHCN screener between 2003 and 2006. Six years after the KiGGS baseline survey, half of the 509 participants who had screened positive (50.6%; 95% CI 44.4-56.8) stated that they were still chronically ill or faced health impairments. In contrast, one fifth of the participants who had provided no evidence of a chronic illness at the time of the KiGGS baseline survey (21.1%; 18.9-23.4) stated that they were now chronically ill or faced health impairments.

Adolescents with a chronic illness reported being chronically ill in young adulthood more frequently than younger children (OR 1.8; 1.02-3.3). Bronchial asthma among 12- to 17-year-olds who had screened positive is associated with a reporting of chronic illness or health restrictions in later life (OR 3.5; 1.6-7.6). Regardless of age, obesity, the presence of asthma or an ADHD diagnosis, chronically ill boys who report pain, and chronically ill children from families with a low socio-economic status and few personal resources (personal protective factors), are at particular risk of a chronic illness in young adulthood.

## 1. Background

In recent years, the burden of illness in childhood and adolescence has shifted away from acute and infectious diseases to chronic diseases [1]. Throughout the world, there has been a sharp rise in the incidence of chronic diseases in childhood [2]. Although the survival chances of premature babies [3] and children with malformations, cancer and cystic fibrosis [4] have improved, today, children and adolescents are more frequently affected by allergic diseases [5], obesity [2], and mental, behavioural and developmental disorders [6]. The burden of disease caused by chronic illness in childhood and adolescence not only affects health in the narrow sense of the term; it is also expressed through the negative impact it has on educational attainment and the way in which it hampers people’s ability to participate in society. In addition, further problems may arise if children and adolescents are stigmatised because of their illness. In Germany, the current prevalence of chronic illness is 16.2% among 0- to 17-year-olds [7]. These changes to the spectrum of disease that affects children and adolescents have been described as the ‘new morbidity’ [8].

The patterns followed by chronic paediatric disorders are more varied than those that occur among adults and often include diverse rare diseases [9]. This makes it difficult to use information about specific medical diagnoses to provide a description of chronically ill children as a whole. As such, ‘generic’ instruments, in other words, tools that are applicable to a wide range of diseases, are more appropriate when studying children who are at particular risk of unfavourable physical or psychological developments due to chronic health problems and who require special health care, therapeutic or general support [10, 11]. One such tool is the CSHCN screener (Children with Special Health Care Needs Screener), which was employed in the baseline study of the German Health Interview and Examination Survey for Children and Adolescents (KiGGS). The CSHCN screener helps to identify children with chronic physical and mental conditions – regardless of the diagnosis in question – through their shared characteristics, such as the increased need for medical care, psychosocial or pedagogical support, and the presence of developmental and behavioural problems that require treatment [12-15]. The CSHCN screener could be used as part of this study because the KiGGS baseline survey obtained data on chronic illnesses directly via information provided by the parents. By the time that KiGGS Wave 1 was conducted, the participants had already reached adulthood, which enabled them to be interviewed directly as part of the telephone survey; a different conceptual approach was employed with regard to the operationalisation of chronic disease [9] and resulted from directly requesting a subjective assessment of whether the respondent felt chronically ill or was facing health-related limitations [16-18].

Beyond the specifics of certain chronic diseases in childhood and adolescence, a generic operationalisation ultimately offers the opportunity to explore the factors that lead to a favourable or unfavourable prognosis. This also particularly enables unfavourable prognoses to be identified among vulnerable sections of the population.

Very few studies of the prognosis of chronic diseases in children have been based on a generic operationalisation [19, 20]. The authors of a longitudinal study from Australia who used a ‘generic’ approach for operationalisation underline the importance of the increased health care needs that occur over a long period of time when interpreting their findings [20]. The present longitudinal analysis employs data from the KiGGS cohort to examine the odds that chronically ill children and adolescents have of no longer feeling chronically ill or facing health impairments in young adulthood; it also studies the factors that prove unfavourable during the transition from childhood to young adulthood.

## 2. Method

### 2.1 Study population

The analysis is based on a data subset sourced from the first two survey waves of the KiGGS cohort. The KiGGS cohort is the longitudinal component of the KiGGS study [21], which is carried out by the Robert Koch Institute in the context of health monitoring. The KiGGS baseline survey is a population-based survey that was conducted at 167 sample points between 2003 and 2006 as a combined interview and examination survey of 0- to 17-year-old children and adolescents (n=17,641; response rate=66.6%) [22, 23]. To ensure the representativeness of the study, a weighting factor was applied that corrected the sample for deviations from the population structure (as of 31 December 2004) in terms of age, gender, region, citizenship and parental education [22]. The persons who took part in the KiGGS baseline survey are continuously monitored into adulthood within the context of the KiGGS cohort. The first follow-up of these participants took place within the scope of KiGGS Wave 1 (2009-2012) as a telephone interview; everyone who had participated in the baseline survey and had stated that they were willing to participate again – all of whom were now aged between 6 and 24 – was invited to do so [24]. Participants aged 11 or above were interviewed directly, and the parents of all under-18s were also interviewed. The response rate was 68.5% (n=11,992). The rate was higher among children and adolescents (72.7%) than among adults (59.9%). The KiGGS sample is corrected for deviations from the population structure. In addition, in order to continue benefiting from the representative sample despite the problem of ‘dropouts’ (study participants who do not participate in later waves), a longitudinal average weight was calculated that adjusted the data set for deviations from the population composition at the time the KiGGS baseline survey was conducted and for the various likelihoods of respondents participating again [24].

**Figure 1:**
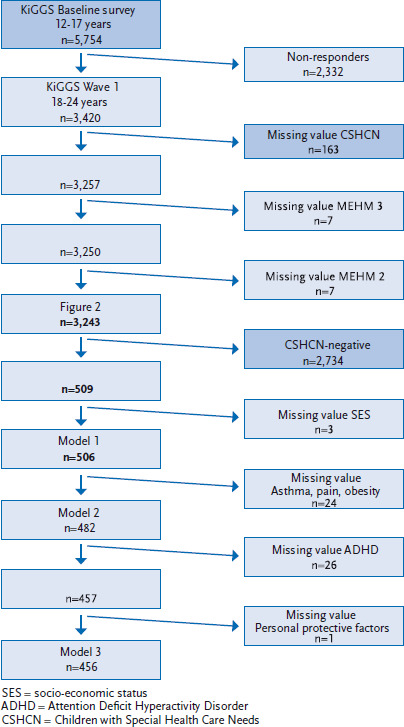
Development of the sample Source: KiGGS Baseline Survey 2003-2006 and KiGGS Wave 1 2009-2012

Participants were included in the analysis set out in this paper if they had reached adulthood at the time of KiGGS Wave 1 and had been at least 12 years old when the KiGGS baseline survey was undertaken. The latter age restriction ensures that the full range of ages covered by the cohort is included in the analysis. Participants with missing answers linked to the variables under assessment were excluded from the analyses (Figure 1).

### 2.2 The operationalisation of chronic disease

In order to identify chronically ill children, the KiGGS baseline survey used the German translation of the CSHCN screener during a written parent questionnaire. This formed part of a generic approach that applied regardless of the diagnosis in question [12, 13, 25]. The screener consists of five main questions about a person’s uptake and needs for special medical or non-medical services (medically prescribed medicines, medical, psychological or pedagogical treatment, advice and support), as well as the presence of functional impairments in a person’s everyday life, emotional and developmental needs and behavioural problems. In each case, two sub-questions are asked about whether an impairment or need is based on a health or developmental problem, whether it has existed for at least 12 months, and, if not, whether it is foreseeable that it may do so. Children were screened as positive if their parents answered ‘yes’ to at least one of the main questions, and the respective sub-questions. The CSHCN screener has a sensitivity of about 80% in clinical studies [9, 12]. A CSHCN screener validation study, conducted in the context of the European DISABKIDS study on chronic diseases in childhood, found that 80% of children who are diagnosed as chronically ill in primary paediatric practices demonstrate a need for care when assessed using the CSHCN screening instrument [9, 15].

In the KiGGS baseline survey, 91% of the study participants provided complete answers to the main and sub-questions. Missing information was mainly due to a lack of German language skills among some participants [26].

In KiGGS Wave 1, generic queries about chronic illnesses were undertaken by asking the respondents – now adults – two questions from the Minimal European Health Modules (MEHM). In selecting an applicable instrument for the study of young adults, the focus was placed on ensuring the results would be comparable with those of other national [27] and international surveys [16]. The questions asked were: ‘Do you have one or more long-lasting, chronic illnesses or health problems?’ This question could be answered with ‘yes’ or ‘no’ and was supplemented by the following statement: ‘Chronic diseases are long-lasting diseases that require regular treatment and monitoring, such as asthma, epilepsy, diabetes or heart disease’. Data was gathered on disease-related impairments using the question: ‘To what extent does a disease lead you to face permanent impairments to your everyday activities? We are referring to periods of at least half a year’. For the analyses presented in this paper, the categories ‘Significantly restricted’ and ‘Restricted, but not significantly’ were grouped and compared with the category ‘Not restricted’. The variable ‘chronically ill or health restricted’ was developed from these two items (question complexes) to analyse the prognosis of chronic illnesses and for the logistic regression analyses. This approach ensured that the operationalisation of KiGGS Wave 1 remained aligned as closely as possible to the CSHCN screener used in the KiGGS baseline survey. The baseline survey screened children as CSHCN positive if an affirmative answer was provided to the question of whether they faced health restrictions that prevented them from doing things that most children of the same age could do, and if these restrictions lasted for at least 12 months. The instruments employed in KiGGS Wave 1 and the baseline study, therefore, differ fundamentally in their conceptual approach. Whereas classification via the CSHCN screener explicitly requires that the specified criteria be present for a long period of time and are based on the consequences of illnesses such as functional and participatory disorders as well as an increased uptake of health care services, the MEHM questions are solely based on a subjective assessment of a disease or condition being described as chronic. When interpreting the following results, therefore, it is important to remember that they should not be understood in terms of the persistence (duration) or the incidence (occurrence) of chronic illness, but as providing information about whether young adults subjectively assess themselves as chronically ill.

In the following, a ‘chronic illness’ is assumed to have been identified if, at the time of the KiGGS baseline survey, a child was screened CSHCN positive, or if information was provided detailing a chronic illness or a health-related impairment during KiGGS Wave 1.

### 2.3 Determining the indicators of chronic illness in young adulthood

The selection of the factors (determinants) from the KiGGS baseline study that act as indicators of chronic illness in young adulthood was explorative and aimed at including both common physical and mental illnesses as well as possible risk factors. In line with the validation sample of the CSHCN screener [12], the KiGGS baseline survey demonstrated that bronchial asthma and attention deficit disorder ADHD – in addition to allergic rhinitis (hay fever) and atopic dermatitis – were the most frequently cited medical or psychological diagnoses among 11- to 17-year-old participants who had screened positive (data not shown). Therefore, these two common childhood physical illnesses (bronchial asthma) and mental disorders (ADHD) were included in the models. The investigation of whether chronically ill children and adolescents with increased body fat (obesity) have a higher risk of reporting that they are chronically ill in adulthood, and whether reported (acute or chronic) pain (as a possible indication of the severity of a disease) is associated with a reporting of a chronic disease is also a reflection of the exploratory nature of the analysis. In line with an understanding that protective factors can mitigate the impact of risk factors, and because the data from the KiGGS baseline survey [28] show an association between these factors and certain chronic diseases, such as ADHD and obesity, in this paper we analysed associations with low levels of personal protective factors. Other possible influencing factors included age, gender and the socio-economic status of the parental home as important socio-demographic characteristics. The following describes the variables that were included from the KiGGS baseline survey in more detail.

#### 2.3.1 Diagnosis of bronchial asthma as a physical disease

Data on the presence of asthma over the last 12 months and/or the use of asthma medication were collected using a computer-assisted medical interview conducted with the parents of children who had been diagnosed with asthma [29].

#### 2.3.2 Diagnosis of ADHD as a mental disorder

Parents of children aged 3 or above were asked: ‘Has your child ever been diagnosed with an attention disorder/hyperactivity?’ Confirmation of a medical or psychological diagnosis was sought using the question: ‘If so, who was it diagnosed by?’ [30].

#### 2.3.3 Pain as a possible indicator of the severity of a disease

In order to gather data on the three-month prevalence of pain, a self-administered written questionnaire was provided to 11- to 17-year-old children and adolescents who were asked: ‘Have you experienced any pain in the last three months?’ [31]. The question could be answered with ‘yes’ or ‘no’.

#### 2.3.4 Obesity as a physiological risk factor

Body size and weight were measured in a standardised manner at an examination centre [32]. Body mass index (BMI: calculated as weight (kg) divided by height squared (m^2^)), was used to identify overweight and obesity. The present paper categorises children and adolescents as obese if they have a BMI above the 97^th^ age- and gender-specific percentile of the reference population. The reference values developed by Kromeyer-Hauschild et al. [33, 34] were used for the evaluations presented in this study.

#### 2.3.5 Personal resources as a protective factor

Personal protective factors are characteristics that are present among the children and adolescents themselves. Examples include a sense of coherence expressed as a feeling of trust that the demands placed upon them during their life are understandable, can be mastered and have a purpose; as well as ‘dispositional optimism’ – a form of general confidence that things will develop positively; and, finally, a general experience of self-efficacy – the belief that they have the necessary competencies to deal with the various situations that they face. Data on personal protective factors were gathered in the KiGGS study from 11- to 17-year-olds using a pre-test consisting of a five-item scale [35]. The respondents were able to provide one of a four-step answer ranging from ‘untrue’ to ‘completely correct’ [36]. The answers are coded so that higher values stand for greater levels of the corresponding resources. The scores were summed and converted to values on a scale between 0 and 100. Once the distribution of responses identified in the KiGGS sample had been taken into account, cut-off values (tolerance values) were determined and the figures gained were categorised as ‘normal’, ‘below average or borderline’ and ‘significant deficit’ (62.6-100=normal; 50.1-62.5=borderline; and 0-50.0=significant deficit). The categories ‘significant deficit’ and ‘below average or borderline’ were grouped for the analyses carried out in this paper [36].

#### 2.3.6 Socio-economic status of the parental home

Social background was included by assessing the socio-economic status (SES) of the parental home at the time the KiGGS baseline survey was conducted. Data on the SES of the parental home were gathered using a multi-dimensional, aggregated index based on the sum of a score developed from the information provided by the parents about their educational and occupational qualifications, occupational status, and needs-weighted net household income. The index enabled the respondents to be categorised into low, medium and high socio-economic groups [37].

### 2.4 Statistical evaluation

The focused sample was examined to check whether longitudinal weighting could compensate for the possibility of systematic non-response for all variables included in the analysis. Systematic self-selection could lead to biased results if, for example, children with chronic illness or risk factors associated with persistent health disorders were to participate less frequently in the follow-up study as this would lead to an underestimation of the proportion of children and adolescents who view themselves as chronically ill in young adulthood. In order to largely exclude biases such as these, the frequencies calculated for the focused sample – in other words, the group of returnees who participated in KiGGS Wave 1 – has to be comparable with the longitudinal weighting of the frequencies gained for the total group of 12- to 17-year-olds from the baseline survey. The respective prevalences led to calculations of 95% confidence intervals (95% CI) and are compared in Table 1. A significant difference was defined as a case in which a 95% CI did not overlap.

Correlation analyses of possible determinants that indicate chronic illness in young adulthood were performed using multivariate logistic regression models. Odds ratios (OR) and the associated 95% CI were calculated, and should be interpreted as ratios of probability. These ratios indicate the factor by which the odds of an indicator occurring in one group is higher or lower than the odds of the same indicator occurring among a reference group. For the sake of clarity, instead of referring to the odds of being chronically ill or the odds of having health-related impairments, the following discusses the risk or chance of being chronically ill or facing health-related restrictions, despite the fact that, strictly speaking, these provide imprecise measurements. The calculation of 95% confidence intervals and p-values was performed in weighted analyses using the survey procedure set out in STATA 14.1SE (StataCorp LP, College Station, TX).

## 3. Results

Of the 5,754 12- to 17-year-olds in the KiGGS baseline survey, a total of 3,420 participated in KiGGS Wave 1 (59.4%). 163 participants were excluded from the analysis because their parents had provided no information for the CSHCN screener (Figure 1). The repeat participants were more likely to be female, from families with high SES, and less likely to be obese or diagnosed with ADHD than the overall KiGGS baseline survey sample. These differences were largely offset by the weighting applied in the analyses (Table 1). Longitudinal weighting, however, did not fully account for the distribution of the lifetime prevalence of medically diagnosed cases of ADHD, but this was achieved with regard to overlapping confidence intervals: the prevalence in the KiGGS baseline sample was 6.5% for 12- to 17-year-olds (95% CI 5.7-7.5), the prevalence from the KiGGS baseline study was 5.0% (4.0-6.2) among the returning participants aged between 12 and 17 (Table 1). In the sample included in the analysis, the proportion of girls was 49.4% (48.6-50.2). The average age was 14.6 (14.5-14.6), and 15.2% (14.1-16.3) of children and adolescents aged between 12 and 17 at the time of the baseline survey screened positive (chronically ill); 4.2% (3.7-4.8) had health-related limitations (Table 1).

### 3.1 Longitudinal follow-ups of chronically ill children and adolescents

**Figure 2:**
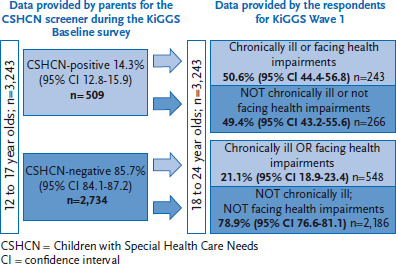
Follow-up observation of chronically ill children and adolescents in young adulthood Source: KiGGS Baseline Survey 2003-2006 and KiGGS Wave 1 2009-2012

Six years after 509 children and adolescents (50.6%, 44.4-56.8) had screened positive in the KiGGS baseline study, half – now aged between 18 and 24 – reported that they were chronically ill or faced health-related impairments. Of the 2,734 children and adolescents who had neither screened positive during the KiGGS baseline study nor provided any indication of a health impairment that pointed to a chronic disease, one fifth (21.1%, 18.9-23.4) reported that they were chronically ill or faced health impairments (Figure 2).

### 3.2 Determinants that indicate chronic illness in young adulthood

**Figure 3:**
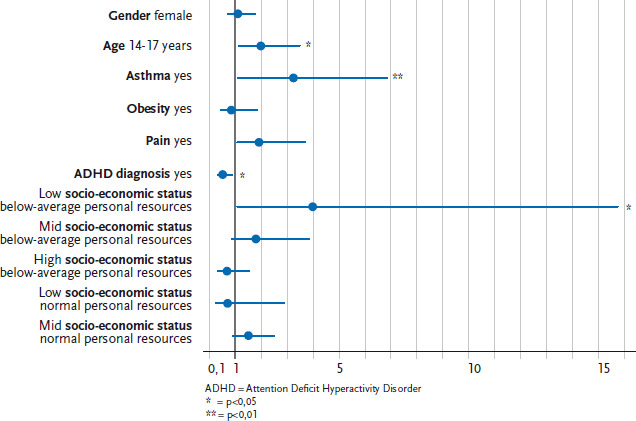
Logistic regression model for the chance that chronically ill 12- to 17-year-olds between the ages of 18 and 24 will self-report as chronically ill. Interaction of socio-economic status and personal protective factors Source: KiGGS Baseline Survey 2003-2006 and KiGGS Wave 1 2009-2012

The risk of young adults reporting a chronic illness after having been screened as chronically ill six years earlier due to data provided by their parents is nearly twice as high among children aged between 14 and 17 as it is for children aged between 12 and 13 (OR 1.8, 1.02-3.3; Table 2). No significant gender differences were identified from the multivariate model. Among both genders, a diagnosis of ADHD is associated with a reduced risk of self-reporting a chronic disease in young adulthood. A significant association exists between current asthma among 12- to 17-year-olds and a reporting of chronic disease six years later (OR 3.49, 1.6-7.6; Table 2). Models that treated gender separately (Table 2) show that this relationship is much more pronounced among boys than girls. Among boys, there is a significant relationship between a reporting of pain in the three months prior to the KiGGS baseline study and a reporting of a chronic illness in young adulthood. This relationship was not identified among girls. The presence of a significant interaction (p=0.033) indicates that a family’s socio-economic status acts as an ‘effect modifier’ on the association between personal protective factors and the reporting of a chronic disease; in other words, the impact of one risk factor (low levels of personal protective factors) is dependent on the presence of another risk factor (low levels of socio-economic status). Although no protective relationship between personal protective factors and the risk of reporting a chronic illness in young adulthood was found for the entire sample (OR 1.3; p=0.4; Table 2), low levels of personal resources among chronically ill children from families with low levels of socio-economic status are a relevant predictor of chronic disease in young adulthood (Figure 3).

## 4. Discussion

### 4.1 Key results

One in two children who screened as chronically ill between the ages of 12 and 17 report a chronic illness in young adulthood; this is the case with one in five children and adolescents who screened negatively. Thus, the onset of chronic disease in young adulthood can often be traced to childhood, and chronically ill children and adolescents are at a greater risk of suffering from chronic illnesses and health impairments in later life compared to their healthy peers. When a chronic illness is identified among 12- to 17-year-olds due to information provided by the parents, children aged between 14 and 17 have a higher risk of reporting a chronic illness in young adulthood than children aged between 12 and 13. Finally, whereas children with bronchial asthma are at a higher risk of reporting a chronic illness or that they face health impairments in young adulthood, children and adolescents screened as chronically ill due to ADHD are at a lower risk of doing so.

Models that treat gender separately show clearer associations among boys, both with regard to age and bronchial asthma. Moreover, a higher risk of developing a chronic disease in young adulthood was identified among boys who report pain, and whose family is classed as being of ‘middle’ socio-economic status. This is not the case with girls.

The risk factors linked to chronic illnesses among 12-to 17-year-olds not only differ between the genders, but also according to the socio-economic status of the child’s family. Low levels of personal protective factors are only associated with a higher risk of self-reported chronic illness among children and adolescents from families with a low socio-economic status.

### 4.2 Discussion of individual relationships

#### 4.2.1 ADHD

This study shows that children and adolescents who have been screened as chronically ill and previously diagnosed with attention deficit/hyperactivity disorder have a lower risk of reporting a chronic disease in young adulthood than those without such a diagnosis. There are a number of points to consider when interpreting this result. The prevalence of a previous diagnosis of ADHD is four times higher among chronically ill boys than among chronically ill girls (data not shown; boys: 33.1%, 95% CI 24.6-42.8; girls: 8.1%, 4.2-14.8). As such, ADHD needs to be included in the model calculation in order to ensure that gender is not affected by biased estimators. In the past, ADHD was considered a disorder of childhood and adolescence; however, remission only occurs in about one third of cases [38]. As such, ADHD is now regarded as a lifelong mental disorder [38] in which the severity of the symptomatology tends to reduce during the transition to adulthood [39]. Moreover, there is often a shift from hyperactivity and impulsiveness to attention deficits (daydreaming, difficulty concentrating, and a lack of ability to organise everyday life) [40]. Whereas children affected by ADHD are usually physically restless, hyperactivity in adulthood is often only present as a form of inner restlessness. Importantly, this shift in symptoms could mean that young adults with ADHD are less likely to perceive themselves as ‘chronically ill’ or as facing ‘health impairments’.

In addition, two methodological factors will have contributed to the lower risk of reporting a chronic illness in adulthood for children and adolescents diagnosed as having ADHD. For example, the CSHCN screener explicitly queries emotional, developmental and behavioural problems. In contrast, KiGGS Wave 1 queried chronic illness among young adults using a question from the MEHM that does not explicitly mention mental health problems and merely lists physical illnesses in the examples it provides (‘asthma, epilepsy, diabetes or heart disease’). As such, participants are likely to assume that ‘chronic illness’ only refers to physical illnesses, leading mental disorders to be underreported.

Finally, children and adolescents who, as part of the KiGGS baseline study, reported that they had been diagnosed with ADHD were less likely to participate in the KiGGS Wave 1 follow-up as adults. Although weighting largely corrected the sample for non-response, it is possible that children and adolescents with severe cases of ADHD and, as such, a higher risk of a worse prognosis [39] did not participate in the follow-up. These factors would lead to a disproportionate number of less strong cases of ADHD (with a higher chance of remission), and this could have led the ‘protective’ relationship of ADHD and the later onset of chronic disease to be overestimated.

#### 4.2.2 Asthma

Childhood bronchial asthma is one disease that is often reversible, but which also typifies chronic conditions. Epidemiological studies have identified a wide range of remission rates for childhood asthma depending on the length of time that the condition was tracked, the age of onset, the study methods employed, the way in which a diagnosis was made, and the severity of the condition [41]. Our analyses demonstrate a 3.5-fold increased risk of children and adolescents with a diagnosis of asthma reporting a chronic illness in young adulthood. However, the confidence interval for this estimator is relatively wide. In the context of the results of specific longitudinal studies on asthma persistence [42], however, this is a plausible result, as the group of children with an asthma diagnosis tracked by KiGGS includes conditions with different degrees of severity, and, as such, a wide range of possible remission rates [41].

#### 4.2.3 Pain

No increased risk of chronic illness in adulthood was found among the group as a whole in terms of self-reported pain during the three months that preceded the study. However, systematic testing revealed a significant effect-modifying impact with regard to gender on associations with pain (i.e., the effect of one risk factor depends on the presence of the other factor). This is particularly clear once the results have been stratified according to gender: whereas there is no association between pain and a later indication of a chronic illness among girls, an indication of pain among boys comes with a fourfold risk that they will report a chronic illness in young adulthood. There are a number of factors that could explain this result. In general, girls report pain much more frequently than boys. However, 17% of 11- to 17-year-old girls report menstrual pain as the main pain that they experience [31]. The high rate of menstrual pain could explain the weaker association between reports of pain and later chronic illness in girls. It is also possible that girls have a better awareness of symptoms of pain and therefore report less severe or short-term episodes of pain more often than boys. This would lead boys to be more affected than girls by the same symptoms. Sensitivity analyses demonstrate that this association persists when the definition of an indication of pain is narrowed to pain that has existed for more than three months.

#### 4.2.4 Personal protective factors

No significant association was identified between personal protective factors and a later indication of chronic disease among chronically ill children and adolescents. However, the analysis of the interaction between socio-economic status and personal resources demonstrates that a lack of personal resources among chronically ill children from low-SES families in adulthood is associated with an increased risk of reporting a chronic disease. Despite the relatively small number of cases, a strong and significant correlation was identified in this case. Thus, personal resources are an important protective factor for chronically ill children from families with low levels of SES.

The targeted promotion of life skills and personal resources among chronically ill children are important measures that promote opportunities for action and the development of coping strategies; this is also the case among children and adolescents with chronic illnesses. Personality traits such as optimism, self-efficacy, and a sense of coherence [43] as well as successful coping strategies [44] are associated with higher levels of therapy adherence and better health [45]. If it is assumed that higher levels of personal resources lead to more effective self-management [46-48], these findings are consistent with studies that demonstrate the importance of good levels of self-management in terms of better disease progression/disease control [49] and those that describe useful approaches to intervention [45]. The significance of low levels of personal resources with regard to the possibility of a chronic illness lasting into young adulthood, especially among children and adolescents from socially disadvantaged families, is in line with the understanding that measures aimed at strengthening self-regulation skills are particularly important for certain groups of chronically ill adolescents [50]. The national health goal ‘Growing up healthy’ underlines the promotion of life skills (the development of psychological strengths and resources), alongside diet and exercise, as key if children and adolescents are to grow up healthily [51].

### 4.3 Strengths and limitations

As part of the follow-up to the first population-based study of the health of children and adolescents, the KiGGS cohort is one of the few longitudinal studies that tracks an entire group of chronically ill children within a specific population. The KiGGS cohort’s wide age range means that data from the first follow-up interviews conducted for a sub-sample already provide information about the development of chronic illnesses and health-related functional impairments that occur during the transition to adulthood and thus enable risk groups to be identified within the coverage provided by the CSHCN screening tool.

This study describes and analyses the presence of a chronic illness during the course of a respondent’s life (the study’s outcome). It used data from the KiGGS baseline survey and KiGGS Wave 1, and both operationalise chronic disease differently. In the KiGGS baseline survey, information about the possible existence of a chronic disease was obtained via information provided by the parents, which meant that it was possible to use the CSHCN screener. In contrast, as the participants had reached adulthood when KiGGS Wave 1 was conducted, data was gathered for this study via telephone interview. The methodological studies conducted on the operationalisation of chronic disease demonstrate that different instruments not only lead to different prevalences, but also to the identification of different groups of chronically ill patients [52-55]. As the CSHCN screener directly collected data on emotional, developmental and behavioural problems, mentally ill children and adolescents are better covered by the screener than through the MEHM question on long-term chronic diseases or health impairments that was used for KiGGS Wave 1. Furthermore, it is likely that the MEHM question particularly favours chronically ill patients who are suffering from one of the diseases stated in the examples listed in the question. This may have led the respondents to treat other diseases and disorders, such as ADHD and obesity, as non-chronic diseases and these would therefore have gone underreported.

As such, the analyses presented in this study do not describe factors that influence the persistence of chronic illness; rather, they identify factors that influence children and adolescents – who have been identified by the CSHCN screener as having a chronic illness – to report a chronic illness in young adulthood. The subjective assessment of general health and chronic disease are important indicators that have become established in health surveys of adolescents and young adults [56]. In cases where the sensitivity and specificity of two instruments differ during investigations of determinants, however, changing the instrument would also affect the identification of risk factors. This issue, therefore, needs further research.

Neither the CSHCN screener nor the MEHM questions differentiate between individuals with chronic diseases who have a high or low chance of temporary improvement (remission) or even complete recovery, nor do they enable differentiation according to the severity of an illness [57]. Furthermore, these instruments do not consider the particular aspects of the prognoses of children affected by multi-morbidities (multiple diseases) or comorbidities (accompanying illnesses). Analyses of the life courses of chronically ill children and adolescents using generic instruments, therefore, cannot replace follow-up studies that focus on specific diseases or disease groups.

## 5. Conclusion

A large proportion of young adults with chronic health problems can be identified as early as childhood or adolescence. The risk of chronic health problems, even in adulthood, is higher for chronically ill adolescents than for chronically ill children; for example, regardless of age, there is a higher risk of being affected by bronchial asthma. Moreover, chronically ill boys who report pain and chronically ill children from families with a low socio-economic status and low levels of personal resources are most at risk of chronic illness in adulthood – regardless of age, obesity, asthma or an ADHD diagnosis.

Finally, children and adolescents who report that they are chronically ill and who have been diagnosed with ADHD report less frequently that they are chronically ill in adulthood. More in-depth studies are needed on this issue in order to differentiate between potential method-related effects, remissions and a possible lack of awareness of ADHD-related symptoms as constituting a chronic disease.

## Key statements

- Half of the children and adolescents who were positively identified as CSHCN during the KiGGS baseline survey went on to state that they were chronically ill or faced health impairments during young adulthood.
- Chronically ill adolescents more frequently report being chronically ill during young adulthood than younger children.
- The factors that are associated with a more frequent reporting of a chronic illness in young adulthood differ in terms of gender and the socioeconomic status of the child’s family.
- Among the children who were positively identified as CSHCN, two groups are most likely to report a chronic illness in young adulthood: boys who state that they are in pain and children from families with a low socio-economic status and very few personal protective factors.
- A generic operationalisation enables a chronically ill child’s development to be followed through longitudinal studies such as the KiGGS cohort. This applies regardless of the specificities of a particular chronic condition. However, the change made to the survey instrument employed, as is the case in KiGGS Wave 1, has made it more difficult to interpret the results.

## Figures and Tables

**Table 1:** Description of the samples Source: KiGGS Baseline Survey 2003-2006 and KiGGS Wave 1 2009-2012

Variables	KiGGS baseline study (2003-2006) 12- to 17-year-olds n=5,298*	KiGGS Wave 1 (2009-2012) 18- to 24-year-olds, analysis sample n=3,243^**^
	% (95% CI)	% (95% CI)
**KiGGS Baseline study**		
**Gender**		
Female	49.4 (48.6-50.2)	50.9 (49.2-52.6)
Male	50.6 (49.8-51.4)	49.1 (47.4-50.8)
**Mean age (years)**	14.6 (14.5-14.6)	14.6 (14.5-14.6)
**Age group**		
12-13 years	30.6 (29.5-31.7)	29.8 (28.2-31.5)
14-17 years	69.4 (68.4-70.5)	70.2 (68.5-71.8)
**Socio-economic status**		
High	16.5 (15.0-18.1)	17.9 (15.8-20.2)
Medium	63.5 (61.8-65.2)	62.5 (60.3-64.7)
Low	20.0 (18.4-21.7)	19.6 (17.8-21.5)
**CSHCN screener**		
Positive	15.2 (14.1-16.3)	14.3 (12.8-15.9)
Negative	84.8 (83.7-85.9)	85.7 (84.1-87.2)
**Health restricted for at least 12 months**		
Yes	4.2 (3.7-4.8)	4.0 (3.2-5.0)
No	95.8 (95.2-96.4)	96.0 (95.0-96.8)
**Asthma**		
Yes	4.5 (3.8-5.3)	4.1 (3.4-5.1)
No	95.6 (94.8-96.2)	95.9 (94.9-96.7)
**Obesity**		
Yes	8.7 (7.8-9.8)	8.1 (6.7-9.6)
No	91.3 (90.2-92.2)	92.0 (90.4-93.3)
**Pain in the last three months**		
Yes	79.5 (78.1-80.9)	79.5 (77.5-81.4)
No	20.5 (19.1-21.9)	20.5 (18.6-22.5)
**ADHD diagnosis at any point**		
Yes	6.5 (5.7-7.5)	5.0 (4.0-6.2)
No	93.5 (92.6-94.3)	95.0 (93.9-96.0)

**KiGGS Wave 1**		
**Chronically ill (MEHM 3)**		
Yes	21.2 (19.1-23.5)	
No	78.8 (76.6-80.9)	
**Health restrictions (MEHM 2)**		
Yes	12.3 (10.7-14.0)	
No	87.7 (86.0-89.3)	
**Chronically ill or health restrictions**		
Yes	25.3 (23.1-27.6)	
No	74.7 (72.4-76.9)	

CI = confidence interval

CSHCN = Children with Special Health Care Needs

MEHM = Minimal European Health Module

ADHD = Attention Deficit Hyperactivity Disorder

* Total group of 12- to 17-year-olds with parental data on CSHCN weighted by the weighting factor from the baseline survey in order to ensure representativeness (weighted on the population as of 31 December 2004)

** Group of repeat participants of people who were aged between 12 and 17 at the time of KiGGS Wave 1; weighted with the longitudinal weight to compensate for the different chances of participating again and to correct for deviations from the population at the time of the baseline survey (31 December 2004)

**Table 2:** Logistic regression models for the odds that chronically ill 12- to 17-year-olds will self-report as chronically ill aged 18 to 24. Longitudinal analyses from KiGGS baseline survey to KiGGS Wave 1 – multivariate model for total sample and stratified by gender Source: KiGGS Baseline Survey 2003-2006 and KiGGS Wave 1 2009-2012

Variables	Total n=456			Girls n=237			Boys n=219		
	OR	(95% Cl)	p-value	OR	(95% CI)	p-value	OR	(95% CI)	p-value
**Gender**									
Male									
Female	1.2	(0.69-2.0)	0.541						
**Age**									
12-13 years									
14-17 years	**1.8**	(1.02-3.3)	0.042	1.7	(0.75-3.8)	0.205	2.3	(0.92-5.6)	0.075
**Socio-economic status**									
High									
Middle	1.6	(0.9-2.7)	0.081	0.95	(0.49-1.9)	0.883	**2.9**	(1.4-6.3)	0.006
Low	1.3	(0.5-3.7)	0.610	1.1	(0.28-4.1)	0.923	1.7	(0.38-7.7)	0.486
**Asthma**									
No									
Yes	**3.5**	(1.6-7.6)	0.002	2.8	(0.93-8.6)	0.068	**4.8**	(1.7-13.5)	0.003
**Obesity**									
No									
Yes	0.9	(0.36-2.03)	0.719	1.3	(0.3-4.9)	0.734	0.81	(0.28-2.4)	0.698
**Pain**									
No									
Yes	1.9	(0.97-3.8)	0.058	0.5	(0.19-1.5)	0.231	**4.3**	(1.7-10.7)	0.002
**ADHD diagnosis**									
No									
Yes	**0.46**	(0.23-0.94)	0.033	0.39	(0.10-1.5)	0.166	0.47	(0.21-1.03)	0.058
**Personal resources**									
Normal									
Below average or clear deficits	1.3	(0.71-2.3)	0.400	1.34	(0.58-3.04)	0.495	1.2	(0.51-3.03)	0.634

Bold: significant (p <0.05) OR = odds ratio

ADHD = Attention Deficit Hyperactivity Disorder

CI = confidence interval
